# Emotional Touchpoints and Emotions of Childbirth: A Systematic Mixed Studies Review and Epistemic Network Analysis

**DOI:** 10.1111/psrh.70022

**Published:** 2025-06-25

**Authors:** Yvonne J. Kuipers, Yvonne Greig, Sandra Atencia Martinez, Maria King, Caroline Hollins Martin

**Affiliations:** ^1^ School of Health and Social Care Edinburgh Napier University Edinburgh UK; ^2^ NHS Lothian Edinburgh UK; ^3^ Organisational Development and Professional Learning University of Leeds Leeds England

**Keywords:** birth, emotional touchpoints, emotions, epistemic, expectations, experiences

## Abstract

**Background:**

Emotional touchpoints are moments during perinatal care that evoke an emotional response in a woman. There is a lack of knowledge regarding the centrality of how touchpoints and emotions are framed in the real‐life experiences of childbearing women.

**Aim:**

To explore how birth‐related emotional touchpoints interact and how the emotions in the context of these healthcare experiences interact.

**Methods:**

We performed a systematic mixed studies review to capture childbearing women's prospective and retrospective reports of birth expectations and experiences. We conducted a literature search in CINAHL (EBSCO), Medline (EBSCO), PubMed, Web of Science, and Ovid, followed by hand searching and forward and backward citation searching from the included articles. We performed a quality appraisal using the Critical Appraisal Skills Program. We used Epistemic Network Analysis to model and visualize the connections and structure of the emotional touchpoints and the emotions.

**Results:**

We included 28 articles, showing overall moderate quality. We constructed two models, one of emotional touchpoints and one of emotions. The emotional touchpoints model showed a strong connection between *Coping* and the *Process of Labor & Birth* and between the *Process of Labor & Birth* and *Beliefs (belief systems) about Labor & Birth*. The primary emotions model showed strong connections between *Joy* and *Fear*, between *Fear* and *Sadness* and between *Sadness* and *Joy*.

**Conclusions:**

This paper illustrates how the multidimensionality of birth‐related emotional touchpoints and the positive and negative emotions prospectively and retrospectively reported by pregnant and postpartum women were distilled—informing the conversation between care providers and childbearing women.

## 1 Introduction

Childbirth is a major life event with the birth functioning as the key transition momentum, being associated with a variety of positive and negative emotions [1]. The United Kingdom National Institute for Health and Care Excellence (NICE) antenatal guideline [2] highlights that the healthcare professional should provide women with practical information about birth and that this information should emphasize aspects such as the onset of labor, preparation for birth, information about pain relief, and the use of a birth plan. Pregnant women find it important to receive information about the birth, but report an overload of information, while postpartum women report that their informational needs are not always met [3, 4].

A touchpoint is a moment during or an aspect of care, for example, receiving information or care practice routines (e.g., blood pressure or temperature check). A negative or positive emotional response connected to this moment or aspect transforms this touchpoint into an emotional touchpoint [5, 6]. In childbirth, emotional touchpoints are often the drivers of the nature of the woman's care expectations, experiences, and perceptions (e.g., good vs bad, positive vs negative) [6] and the memories of the birth experience [7, 8]. Emotional touchpoints can help to identify crucial points during care expectations and experiences of childbearing women, gaining a deeper insight into what is important to women as maternity service users and evaluating care practices [5, 6, 9]. A multitude, dynamic, and dense interactive complexity of emotional touchpoints and childbirth‐related emotions has been reported in the literature, often in the context of the maternity care system, in terms of sequence, causality, predictions, or as isolated components [10, 11, 12]. Additionally, there is an overload of information during antenatal care, not meeting women's information needs, potentially leading to anxiety and stress and contributing to dysfunctional use of the information [3, 13]. The centrality of emotional touchpoints and emotions and how these exist or are framed in childbearing women's real‐life experiences of childbirth could promote meaningful conversations between maternity care providers and women about their expectations or experiences of childbirth, which could help meet women's information needs and their sense of well‐being. Therefore, it could be valuable to identify the emotional touchpoints occurring in the real‐world experiences of childbearing women. This would proactively inform the load, direction and content of the dialogue between the woman and the care provider during antenatal and postpartum care [5, 9]. The real‐life experiences and experiential self‐reports of childbearing women are reliable resources for collecting information on emotional touchpoints [14, 15]. This study explores how birth‐related emotional touchpoints interact and how the emotions in the context of these care experiences interact.

## 2 Materials and Methods

### 2.1 Design

We conducted a systematic mixed studies review, enabling evidence from diverse study designs to provide a comprehensive and detailed understanding of the current base of evidence [16]. Because childbearing women and childbirth are part of a social space of birth, characterized by its unique discourse, phenomena, and (institutional) practices [8], we adopted a social epistemological perspective. Social epistemology studies the properties of groups, subcultures, or social systems, drawing on sources of knowledge obtained from communities or contexts in which the concept or topic of study is situated [17]. Childbearing women are a recognized subgroup in the social system of maternity services [8, 10]. We (1) reviewed primary research from within the literature to capture childbearing women's prospective and retrospective reports of birth expectations and experiences being collected in different methodological ways; (2) identified, synthesized, and analyzed the emotional touchpoints and emotions within these expectations and experiences; and (3) established patterns of connected birth‐related emotional touchpoints and emotions—reported by childbearing (pregnant, laboring, and postpartum) women.

### 2.2 Information Sources and Search Strategy

We searched the following databases (May 2022): CINAHL with Full text (EBSCO), Medline (EBSCO), PubMed, Web of Science Core Collection, Ovid Nursing, and Ovid Emcare. We used the same free text search terms and date, and language limiters as the database searching, to conduct online hand‐searching in LibrarySearch and GoogleScholar and childbirth journals with full text not indexed in any of the databases (August 2022). Publication dates were restricted to anything from 2000 to May 2022 and limited to the English language. We chose 2000 as in response to the normal versus interventionist birth debate, women were more actively encouraged to concentrate on their intrapartum expectations and needs—thus, relevant research was expected to be published from 2000 onwards [18]. We screened the sources found through the database and hand‐searching, and then in November 2022, the librarian (MK) carried out both forward and backward citation searching from the included articles. We checked the reference lists of reviews and sources of citations. Appendix A shows the keyword terms used in the database searches and the full set of database searches and results.

### 2.3 Study Screening and Selection

Papers were included if the following criteria were met:

1. Participants included women who have given birth, women of all parity and modes of birth with a term gestational age of 36 completed weeks, and maternal age of 18 years or older; high and low‐risk women; women who received care from a maternity care provider, either the midwife, obstetrician, or general practitioner; in any healthcare setting, either primary, secondary, or tertiary.
2. Outcome evaluations included measures of expectations and/or experiences of labor and birth, sense of control or empowerment, birth preparedness, choice and/or decision making, pain, coping with pain, mode of birth, satisfaction, fulfillment, and emotions, including fear of birth.
3. Study methods included quantitative or qualitative primary research, including mixed methods studies involving any number of participants.

Exclude:

1. Parenting, motherhood/being‐a‐mother, and breastfeeding as measures of evaluation or outcome.
2. Studies with purposive or selective samples of women with unintended pregnancy.
3. Studies focusing on babies born with expected anomalies/diseases and/or born prematurely.
4. Societal or cultural perceptions, or perceptions of partners or healthcare professionals.
5. Reviews, conference papers, intervention studies/meta‐analyses, discussion papers, protocols, validation studies, and dyads of women and partners in which it was not possible to distinguish between the findings of the women and (birth)partners.

To screen and select the retrieved titles/abstracts, we imported the search results into EndNote X9.3 reference manager software. To establish initial inter‐rater validity, three reviewers (YK, YG, SAM) individually screened the first 100 results. Two reviewers (YK, YG) individually screened the following papers. Once their title and abstract screening were complete, we retrieved the full texts. The screening of the selected papers was individually carried out by YK, YG, and SAM. We scrutinized full texts for (1) reports on emotional touchpoints, that is, the mentioning of care expectations and experiences during the woman's perinatal care (e.g., care management, policies, communication, information provision, decision‐making, and interpersonal (inter)action) attached with emotion(s), and (2) women's reports on emotional adjectives, sentiments, and values. Papers reporting both aspects were included. We discussed and resolved the disagreements.

### 2.4 Data Collection Process and Data Items

We designed data extraction forms for the item to be extracted from the data (e.g., authors, year of publication, design and country of study, sample characteristics and data collection period).

Additionally, we extracted data for the following domains: (1) emotional touchpoints and (2) emotional adjectives, sentiments, and affective values. Each study was individually read in full by YK and YG, and emotional touchpoints and the emotional adjectives, sentiments, and affective values were highlighted in the text and assigned to the relevant domain. YK and YG compared and discussed, reaching a consensus on the findings.

### 2.5 Quality Appraisal of Individual Studies

We used the Critical Appraisal Skills Program (CASP) tool for qualitative and cohort studies for quality appraisal to assess the degree of the total evidence from studies included in the review [19, 20]. To ensure greater consistency with scores from the CASP Qualitative Research tool, we amended the tool for Cohort Studies from 12 questions to 10 (omitting the item on fit with other evidence and the local application of results item, which were not relevant to our synthesis or consistent with other tools) [17, 21]. Three researchers (YG, SAM, CHM) independently assessed the quality of each study, and the scores and findings were discussed until the researchers reached a consensus on the CASP scores. A total score of 20 = high quality; 16–19 = moderate quality; and ≤ 15 = low quality [21].

### 2.6 Organizing the Data

First, we extracted and summarized the study characteristics. Second, we constructed a framework to organize the data obtained from the survey responses in the quantitative studies and the quotes in the qualitative studies. Two researchers (YK, YG) independently read the selected full texts and scrutinized the results sections of the papers, annotating (1) instances where an emotional touchpoint was reported in connection to expectations and reality of birth and (2) instances where emotional adjectives, sentiments, or values associated with expectations and reality of birth were reported. The emotional touchpoints and the adjectives/sentiments/values were often not reported together. Therefore, we constructed two distinct frameworks: one with emotional touchpoints and one with emotional adjectives, sentiments, and values as the two constructs of interest. We extracted the annotated text from the data and added these to the respective frameworks to provide an overview of what was reported in the studies.

We used an inductive approach to produce an overview of the content of the different data. The emotional touchpoints were labeled, bringing together similar pieces of text within a study and across studies—known as coding [22]. This was followed by comparing, grouping and subdividing groups of labels, resulting in overarching content categories within which several content labels were grouped [22]. We developed the content categories during the process of labeling. The labeling of the adjectives, sentiments and values was guided by Plutchik's wheel of emotions (https://www.6seconds.org/2022/03/13/plutchik‐wheel‐emotions/), which consists of eight primary emotions: anger, anticipation, disgust, fear, joy, sadness, surprise, and trust [23, 24, 25]. The wheel can be used for emotion annotation and categorization [25, 26]. Any reported emotion was related to any of the basic emotions from the wheel. The findings were compared, and agreement was reached about the labels and the content categories.

### 2.7 Analysis

We used the Epistemic Network Analysis (ENA) web tool (version 1.7.0) to reduce the dimensionality of the emotional touchpoints and the primary emotions to create a systematic understanding of meaningful features and interactions between emotional touchpoints and between primary emotions. ENA identifies, quantifies, and visualizes the strength of associations between connections in the data, deriving from various data sources and groups or communities accounting for group context—in our case, quantitative and qualitative study findings and childbearing women as the objects of interest [27, 28, 29], ENA includes three parameters: units, conversations, and codes [27, 28]. Each paper served as a unit, the extracted emotional touchpoints and emotions data served as conversations, and the emotional touchpoint content categories and primary emotions served as codes. Binary coding was used where the values were either 1, indicating the code was present in the study, or 0, indicating the code was not present. ENA generates visual interpretations of the interactions among codes to depict the structure and strength of those interactions [27, 28]. We constructed two models: one for the emotional touchpoints and one for the primary emotions. Nodes in the model represent the codes—the features in the dataset. ENA then uses the coded data to generate ENA models based on the co‐occurrence of codes [27, 28]. We used a moving stanza window to accumulate co‐occurrences. By quantifying these co‐occurrences, two weighted models were created in which the edge (or line thickness of the connection between nodes), the node size of a given code, and the node location relative to other nodes all can provide insight into the data. The models can be interpreted such that thicker lines between nodes depict more frequent connections (stronger connections), and nodes positioned closer together have similar co‐occurrence patterns in the dataset [27, 28, 29]. For the dimensional reduction, we used a singular value decomposition, which produces orthogonal dimensions explaining the model's variance in code‐occurrences of the first (*x*‐axis) and second (*y*‐axis) dimensions. Pearson correlation coefficient reports the goodness of fit; the closer the value is to 1, the higher the model's goodness of fit [27].

### 2.8 Protocol and Registration

The protocol was registered on the Prospective Register of Systematic Reviews (PROSPERO) database (CRD42022320325).

## 3 Results

### 3.1 Study Characteristics

**FIGURE 1:**
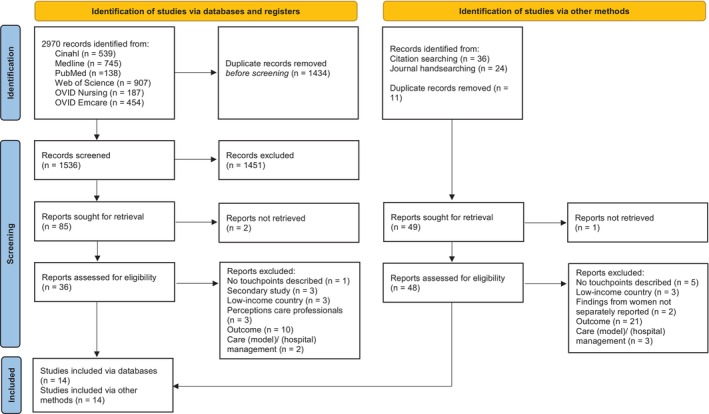
PRISMA flow diagram.

Our searches identified a total of 2970 records. Handsearching and citation searching identified a further 50 studies. After removing duplicates and screening abstracts, titles and full texts, 28 studies remained for inclusion (Figure 1). Decisions between the researchers to include or exclude were concordant in 97.6% of cases. The included studies are summarized in Table 1. Most studies were conducted in Europe (*n* = 15), six in Asia, four in Australia and one in the United States. Three studies were conducted in multiple countries (Belgium and the Netherlands, Scotland, the Netherlands and Switzerland, and Australia and Jordan). Sample sizes ranged from eight to 1042, with an average of 213 and a total number of 6191 participants. Of the 28 studies, 11 included primigravid/nulliparous women, two studies included multigravida/parous women and 15 included samples with both primigravid/nulliparous and multigravida/parous women. The studies were published between 2001 and 2022 (Mean = 2012, Median = 2013), of which 16 were published after 2012. Three periods were identified in the perinatal journey of childbearing women: the antenatal, intrapartum and postnatal period. Nineteen studies collected data in the antenatal period, during all trimesters of pregnancy, but predominantly during the third trimester (with an average of 29 weeks gestation). Two studies included data being collected during early labor and the third stage of labor, and 19 studies included data collected during the postpartum period (between 12 h and 15 years postpartum). Ten studies collected data during more than one phase of the perinatal journey. The 28 studies reported between one to 22 emotional touchpoints, including 248 entries, of which 64 were duplicates and 185 unique emotional touchpoints (most often reported category: “process of labor & birth”, *n* = 95/185). A total of 353 emotional adjectives, sentiments and affective values were reported, varying between two to 38 entries per study, including 169 duplicates and 184 unique adjectives, of which 68/184 were positive (most often reported: joy 19/184) and 116/184 negative adjectives, sentiments and values (most often reported: “fear”, *n* = 23/184).

**TABLE 1:** Overview study characteristics, touchpoints and emotions.

Source	Country of study	Design of study	Aim(s) of study	Sample	Time period data collection	Emotional touchpoint(s)	Emotional adjectives, sentiments and affective values
Akca et al. [30]	Turkey	Prospective study, questionnaire	To assess the influence of a systematic multidisciplinary birth preparation program on satisfaction with childbirth experience and to detect factors that affect the childbirth satisfaction	77 nulli‐ and multiparous women attending the “happy pregnancy program”; 75 nulli‐ and multiparous women in the control group receiving care‐as‐usual	< 48 h postpartum	Communication with staff Involvement/participation in decision making Pain intensity/level Control	Disappointed; Feeling cheated; Exhausted; Painful; Anxious; Enthusiastic; Delighted; Happy; Satisfied; Confident; Relaxed
Aksoy et al. [31]	Turkey	Single‐site study prospective cohort study	To assess the relationship between pain expectation before labor, labor pain and pain perception after the labor	230 pregnant multiparous women from one obstetric unit in a tertiary hospital	At time of admission to hospital in (early) labor, after the 3rd stage of labor and 12 h postpartum	Pain intensity/level Pain expectations Pain perception Memory/recollection of the pain Healthcare provider Complications during birth	Pain; Dissatisfied; Negative; Happiness; Satisfied; Positive
Alehagen et al. [32]	Sweden	Prospective cohort study	To explore the correlations between fear of childbirth during pregnancy and early labor and postpartum	47 nulliparous women	Antenatally (37–39 weeks' gestation), early active labor and 2 h, 2 days, 5 weeks postpartum	Fear of pain Fear of childbirth	Fear; Afraid
Ayers and Pickering [33]	United Kingdom	Prospective study using a survey (postal) questionnaire	To examine (1) the relationship during pregnancy between expectations of birth and symptoms of anxiety; (2) the relationship between expectations and subsequent experience of birth; (3) the effect of parity on expectations and experience	289 pregnant nulli‐ and multiparous women (240 completed questionnaires during pregnancy and 245 postpartum)	36 weeks' gestation and 1 week, 6 weeks, 6 months postpartum	Pain Obstetric event(s) Epidural analgesia Choice of pain relief Control of panic Control of birth position Duration/length of labor	Embarrassing; Challenging; Exciting; Frightening(ed); Traumatic; Enjoyable
Bar‐On et al. [34]	Israel	Questionnaire	To determine whether maternal expectations and predictions regarding mode of delivery and epidural anesthesia aligned with birth outcomes	280 primiparous low‐risk singleton pregnant women in late third trimester of pregnancy	37–41 weeks' gestation	Expectations and predictions mode of birth (vaginal birth vs cesarean) Beliefs (system) about birth Beliefs (system) of the hospital/care provider about birth (Intention) epidural analgesia	Optimism; Pessimism
Bayes et al. [35]	Australia	Qualitative analysis of open answer question of a survey, questionnaire	To investigate women's prenatal and postnatal levels of childbirth fear	141 primi‐ and multiparous postnatal women who had given birth in a public tertiary hospital	6–14 weeks postpartum	Pain relief Physiological birth (Unexpected) complications during the birth (Emergency) Interventions during the birth Attitude of care provider(s) during birth Type of support for care provider(s) during birth Birth environment Safety Informed consent Evaluation/debriefing (coming to terms with what happened)	Painful; Distressing; Negative; Worried; Fearful; Scared; Panic; Concern; Dangerous; Perfect; Fulfilling; Triumphant; Wonderful; Beautiful; Fantastic; Positive; Good; Enjoyable; Confident; Calm; Relaxed; Secure; Safe; Harmed; Horrible; Ordeal; Bad; Frustrating; Complicated; Upsetting; Unexpected
Borelli et al. [36]	United Kingdom	Qualitative (Grounded theory) semi‐ structured interviews	To explore first‐time mothers' expectations of labor and birth, coping strategies they adopt during pregnancy toward childbirth and coping strategies they expect to use during labor and birth	14 first time mothers with uncomplicated pregnancies	3rd trimester of pregnancy	Waiting for the unknown	Comfortable; Uncomfortable; Uncertainties; Doubtful; Ambivalent; Fear; Disappointed
Christiaens and Bracke [37]	Belgium and Netherlands	Self‐reported survey	To examine multiple determinants, the fulfillment of expectations, labor pain, personal control and self‐efficacy, for their association with satisfaction with childbirth in a cross‐national perspective	605 nulli‐ and multiparous women (261 Belgian/344 Dutch)	30 weeks' gestation and < 2 weeks postpartum	Pain experience Personal control Expectations of the birth Planned place of birth Mode of birth Midwife Physician Partner Baby	Unbearable; Bad; In control; Loss of control (of self)
Colciago et al. [38]	Italy	Qualitative (Husserlian phenomenological) study, focus group interviews	To explore first‐time Italian parents' expectations of labor and birth	8 first‐time pregnant women and their partners with a low‐risk and singleton pregnancy	Late 3rd trimester of pregnancy	Unpredictable nature of labor and birth Midwife Partner Coping strategies	Uncertainties; Doubtful; Hopeful; Helpless; Fear of Pain; Fear of labor; Fear of the unknown; Terrified; Scared; Fear; Anxious; Worried; Concerned; Disappointment; Perfect
Dahlen et al. [39]	Australia	Grounded theory, in‐depth interviews	To explore the experiences of a group of first‐time mothers who had given birth at home or in hospital	19 first‐time mothers who had given birth at home, (private) hospital or birth centre	6–26 weeks postpartum	Trust in the midwife Reputation of (individual) care givers and institutions Choosing a caregiver Choosing place of birth Sourcing information about birth Personal beliefs (system) about birth Personal beliefs (system) in ability to give birth Fear of death or dying Preference of route of care (medical vs midwifery) Personal identity as a woman and a mother	Pain(ful); Negative; Distress; Confident; Unconcerned; Okay; Relaxed; Not bothered; Fear; Anxiety; Scared; Very afraid; Afraid; Wary; Dangerous; Love; Positive; Secure; Dependence
Fair and Morrison [40]	North Carolina, United States	Longitudinal prospective exploratory study, orally administered survey	To explore the relationship between perceptions of prenatal control, expectations for childbirth, and experienced control in labor and birth and how they individually and collectively affect birth satisfaction	31 primiparous women	26–40 weeks' gestation and ≥ 6 weeks postpartum	Fear of birth Control Expectations of control Health of mother and baby Mental health/emotions	Unsatisfied; Ignored; Scary; Frightening; Not in control; Fulfilling; Satisfied; In control
Fenwick et al. [41]	Perth, Australia	Qualitative study using an explorative descriptive design, telephone interviews	To explore and describe the labor and birth expectations, and to identify the factors that influence these expectations	202 self‐selected nulli‐ and multiparous pregnant (*n* = 14) and postpartum (*n* = 188) women	12–16 months postpartum	(Changing) circumstances that are beyond own control Duration/length of labor Complications Involvement/participation in birth process Unpredictable or uncontrollable aspects of birth Support during birth (midwife) Decision making Trust in care professional Health baby Horror stories of others Beliefs (system) about birth (being a medical event) Control Coping Choice Interventions Mode of birth Protective mechanisms when expectations not met	Anxious; Scared; Frightened; Not in charge; Not in control; Fear; Dread; Terror; Scary; Worried; Nervous; Difficult; Traumatic; Risky; Painful; Fulfilling; Satisfied; Wonderful; Awesome; Exhilarating; Happy; Achievement; Beautiful; Successful; Proud; Completeness; Womanly; Natural; Normal; Easy; Simple; Fine; Well; Bad; In charge; In control; Calm; Hope
Fleming [42]	Switzerland (4 cantons)	Hermeneutic approach, semi‐structured interviews	To develop a model of the emerging expectations of giving birth and the subsequent experiences of healthy primigravid women	75 healthy primigravidae women with straightforward pregnancies	20–24, 35–37 weeks' gestation, and 6 weeks, 6 months postpartum	Enormity of life change Mode of birth (natural vs cesarean) Getting it right the next time Place of birth Care during birth Expectations about the birth Debriefing the birth Societal beliefs (system) of birth Going to hospital during birth Partner present at the birth Beliefs (system) of the healthcare professional about birth Involvement of healthcare professional during birth	Stupid; Frustrations; Fear; Frightening; Scared; Remarkable; Balanced; Fine; Okay; Intense; Happiness; Positive; Magical; Wondrous; Disappointment; Uncertainty; Trust; Calm; Confident; Bad; Worse; Risk; Dislike; Denial; Battle
Gibbins and Thomson [43]	United Kingdom	Qualitative study using a phenomenological approach, interviews	To explore, describe and understand the expectations during pregnancy and subsequent experiences of childbirth in primiparas	8 pregnant women, expecting their first baby	> 36 weeks' gestation and < 2 weeks postpartum	Duration/length of labor Coping with pain Coping with labor Ideals about labor Midwife present at the birth Partner support during the birth Control Power Pain relief Feeling accepted by staff	Worse than expected; Disappointment; Pain; Unfortunate; Uncertainty; Sense of purpose; Confident; Okay; Intense; In control; Safe; Calm; Fear; Anxious
Hauck et al. [44]	Perth, Australia	Qualitative study using an exploratory descriptive design, in‐depth individual interviews	To explore and describe the influence of childbirth expectations on women's perception of their birthing experience and expectations for subsequent births	20 women, 11 primiparas and 9 multiparas, who between them had experienced 31 births	< 12 months postpartum	Health baby Beliefs (system) about birth (natural vs medical event) Expectations about own involvement and participation Perception of pain Expectations about the birth process Decisions care management/medical interventions Circumstances that are beyond own control Relationship with healthcare professional present at the birth Fears Unpredictable nature of birth	Fulfilling; Rewarding; Beautiful; Positive; Pleasant; Scary; Frightening; Traumatic; Fear; Battle; Anger; Horrible; Upsetting; Negative; Disappointment; Painful; Surprised; Hard; Worse
Hildingsson et al. [45]	Northern Sweden	Prospective longitudinal study	To explore the prevalence of Swedish women reporting a very positive birth experience 2 months and 1 year after childbirth and identify factors associated with this experience. In addition, the study aimed to identify whether women's assessment of their birth experience changed over time	928 primi‐ and multiparous postpartum women	2 months, 1 year postpartum	Beliefs (system) about birth Support midwife during the birth Support partner during the birth Involvement/participation in decision making Controlling the body Midwife present at the birth Information about progress of labor Medical care Pain relief Midwife involving the partner during the birth Debriefing	Positive; Fear
Hildingsson [46]	Northern Sweden	Prospective regional cohort study	To describe pregnant women's expectations of birth and to investigate if these expectations were fulfilled. An additional aim was to determine if unfulfilled expectations were related to the mode of birth, use of epidural and the birth experience	1042 primiparous (*n* = 461) and multiparous (*n* = 575) women	32–34 weeks' gestation and 2 months postpartum	Preferred mode of birth Support from midwife during the birth Support from partner during the birth Involvement/participation in decision making during birth The midwife's presence in the birth room Mental health/emotions Intention epidural analgesia Mode of birth	Anxiety; Fearful; Negative; Less good; Dissatisfied; In control; Good; Better; Positive; Fulfilled
Hussein et al. [47]	Australia and Jordan	Qualitative interpretive study, interviews	To examine Jordanian women's experiences and constructions of labor and birth in different settings (home, public and private hospitals in Jordan, and Australian public hospitals), over time and across generations	27 Jordanian prim‐ and multiparous postpartum women 20 living in Jordan, 7 living in Australia	Up to 15 years postpartum	Place of birth Privacy of birth environment Cleanliness of birth room Labor pain Beliefs (system) about birth being a normal event of life Coping Being a good mother Non‐medicate approach to pain relief Another person being present in the room during birth Support from midwife during birth Mental health/emotions Function of labor pain Vaginal examination Support of birth partner Pain relief Meeting the baby Skin‐to‐skin Bonding with the baby Female/male doctor Number of doctors and/or midwives present during birth Being left alone during labor When not being listened to	Unbearable; Embarrassing; Annoying; Frustrating; Uncomfortable; Nightmare; Surprised; Extraordinary; Painful; Suffering; Negative; Distressing; Regrets; Comfortable; Good
Korukcu et al. [48]	Turkey	Mixed methods: paper and pencil survey and face‐to‐face interviews	To examine the relationship between previous birth experiences and the fear of birth in the current pregnancy	309 multiparous women with healthy pregnancies	28–40 weeks' gestation	Fear of birth Previous birth experience(s)	Fearful; Happy; Proud; Painful
Luyben et al. [49]	Netherlands, Scotland and Switzerland	Grounded theory using semi‐structured interviews	To investigate important aspects of provision of routine antenatal care from women's points of view in the Netherlands, Scotland, and Switzerland and to construct a conceptual model of care during pregnancy, informed by women	17 (5 Scottish, 5 Swiss and 7 Dutch) nulli‐ and multigravida/parous women	8–36 weeks' gestation and 26 weeks, 5 years postpartum	Beliefs (system) about birth (Risk of) having a handicapped child Risk own life Previous experiences birth Differing beliefs about birth than partner Mode of birth Attitude care provider Whom to contact Debriefing birth (Non)closure	Confidence; Perfect; Anxiety; Risk; Negative; Wrong; Unsure; Difficult; Exciting; Wonderful; Worrying
Melender [50]	Finland	Semi‐structured interviews	To describe pregnant Finnish women's perceptions of a good childbirth	24 nulli‐ and multiparous women	10–40 weeks' gestation	Beliefs (system) about birth Place of birth Obstetric interventions (need, rationale, personal stance) Consent/agreement Privacy birth room Hygiene birth environment Lighting birth room Space to move around in birth room/environment Equipment birth room Atmosphere during the birth Normality of birth Optimal duration/length of labor & birth (Voicing) specific wishes Attitude care provider during birth Care providers' personal characteristics Communication style care provider(s) Staying calm	Scary; Frightening; Risk; Fear
Oweis and Abushaika [51]	Jordan	Descriptive study using questionnaires	To explore pregnant Jordanian women's expectations of their first childbirth experience and to determine their expectations of nursing and midwifery support in the intranatal period	77 primigravid women	All trimesters of pregnancy, predominantly the 3rd trimester	Risk & safety/birth as a danger Health baby Treatment/support care provider Privacy during the birth Pain Duration/length of labor & birth Difficulty of labor and birth Coping Losing control Involvement/participation in decision making Relaxation What does the baby look like Baby being harmed Attachment/bonding Surrendering to others' decisions	Full of surprise; Lonely; Strong; Confident; Afraid; Weak; Safe; Independent; Depressed; Tensed; Delighted, Proud; Give in; Quiet; Relaxed; In pain; Enjoyable; Natural; Understandable; Behave badly; lose control; Cry; Tolerate
Penacoba‐Puente et al. [52]	Spain	Longitudinal study	To analyze the change of childbirth expectations over the course of pregnancy as well as their relation to socio‐demographic and clinical variables	285 nulli‐ and multiparous pregnant women receiving obstetric care, mentally and physically healthy	1st, 3rd trimester of pregnancy	(Risk of) having a handicapped child Risk own life Comforted by partner/support partner during birth Routine (medical) procedures Staff listening to concerns Being taken seriously by medical staff Information given about progress of labor Labor pain Support care provider during the birth	Happy; Excited; Relaxed; Embarrassed; Uncomfortable; Worried; Afraid; Panic; Concerned; Scared
Preis et al. [53]	Israel (metropolitan area)	Observational study, self‐administered questionnaires	To assess women's beliefs about birth as a natural and safe or medical and risky process and study the associations of these beliefs with fear of childbirth and planned birth choice	746 nulli‐ and multiparous low and high‐risk women with a singleton pregnancy	2nd, 3rd trimester of pregnancy	Fear of birth Beliefs (system) about birth being normal vs medical Institutional control/permissions	Fear; Worries; Loss of control; suffer
Preis et al. [54]	Israel	Longitudinal prospective study	To test the hypotheses that (1) a more natural mode and place of birth would lead to greater birth satisfaction and incongruence with one's birth plan and actual birth would lead to lower birth satisfaction, (2) perceptions of control would mediate the sociation between the predictors (place and mode of delivery and incongruence with birth plan) and birth satisfaction, and (3) emotions experienced while giving birth and perceptions of intrapartum care would mediate the association between perceptions of control and birth satisfaction	330 primiparous women	29–34 weeks' gestation and 2 months postpartum	Birth environment Medical staff's actions during birth Interventions during birth Own behavior during birth Mode of birth Birth plan Perceptions of care Mental health/emotions Choice of pain relief, including epidural	Fear; Anger; Guilt
Striebich and Ayerle [55]	Germany	Interpretative‐hermeneutical study, problem‐centred interviews	To gain a deeper understanding of the beliefs of women regarding their impending birth and the coping resources they possess to cope with their fear	12 nulli‐ and primiparous pregnant women	21–37 weeks' gestation	Physiological/natural birth Fear of childbirth Own passive role vs active role during labor Mastering the physical process of labor Body's ability to give birth Coping with pain Control Unexpectedness/unforeseeable (extreme life‐threatening) situations Cooperative behavior/cooperation/surrendering/being a good girl‐institutional norms and social norms Complications Technological medicine/technocratic birth Right time to go to hospital Role midwife (support & guidance) Relationship with midwife Standard routines Interventions Pain relief options	Proud; Surprise; Fear; Paralyzed; Stress; Afraid; Tense; Vulnerable; Painful; Guilt; Bad; Catastrophe; At ease
Turan et al. [56]	Turkey	Qualitative in‐depth interviews (interpretive content analysis)	To give voice mothers' expectations of midwives during the birth process, the car received by them, and their views about the delivery environment	15 primiparous women who had a vaginal birth in a public hospital	8–24 h postpartum	Birth room/environment (hygiene, privacy) Prejudices about the midwife's behavior during labor Intensity of the birth process Birth as a life event Mental health/emotions	Good; Happy; Terrifying; Scary; Intense; Fear; Risk; Depressing; Pain; Lonely; Sad; Negative; Bad; cold; Chaotic
Wiklund et al. [57]	Stockholm, Sweden	Comparative study	To study the outcome of labor and women's perceptions of being referred after onset of labor	266 nulli‐ and multiparous women with a 37–42 weeks' uncomplicated pregnancy, fetus presenting by the vertex and spontaneous onset of labor	1 month postpartum	Place of birth Continuity of care Participation in (planning) care during labor Car journey to chosen place of birth Resource limitations – ‘Full house’	Baffled; Despair; Upsetting; Disturbing; Wrong; Anxiety; Stress; Panicked; Nervous; Sad; Afraid; Worried; Security; Safe; Fulfilled; Positive; Friendly; Insecure; Difficult; Disappointed

### 3.2 Study Quality

The CASP critical scores showed a mean score of 16.9/20 (standard deviation 2.9, range 11–20) for the 14 quantitative studies and a mean score of 18.4/20 (standard deviation 2.1, range 12–20) for the 14 qualitative studies. Of the 14 quantitative studies, two demonstrated high quality, eight were moderate, and three were low quality. Three out of the 14 qualitative studies showed high quality, nine showed moderate quality, and two showed low quality. Overall, the 28 studies showed moderate quality [21]. Low quality was due to the unclear or lack of reported validity of the cohort studies and researchers' bias and unclear reports of transferability in the qualitative studies (Table 2).

**TABLE 2:** Quality appraisal.

Author(s)	1	2	3	4	5	6	7	8	9	10	Total score
CASP items Cohort study checklist											
Akca et al. [30]	Y	Y	Y	?	Y	Y	Y	Y	?	?	17
Aksoy et al. [31]	Y	Y	Y	?	N	N	?	?	?	Y	12
Alehagen et al. [32]	Y	?	Y	Y	Y	N	Y	Y	Y	Y	17
Ayers and Pickering [33]	Y	Y	Y	Y	Y	Y	Y	Y	Y	Y	20
Bar‐on et al. [34]	Y	Y	?	?	Y	Y	Y	Y	Y	Y	18
Christiaens and Bracke [37]	Y	Y	Y	Y	Y	Y	?	?	Y	Y	18
Fair and Morrison [40]	Y	N	?	?	?	?	?	?	?	Y	11
Hildingsson et al. [45]	Y	Y	Y	Y	Y	Y	Y	Y	Y	Y	20
Hildingsson et al. [46]	Y	Y	Y	Y	Y	Y	?	?	Y	Y	18
Korukcu et al. [48]	Y	Y	Y	Y	Y	Y	?	?	Y	?	17
Oweis and Abushaika [52]	Y	Y	?	?	?	?	?	?	Y	Y	12
Penacoba‐Puente et al. [55]	Y	?	?	Y	Y	Y	Y	Y	Y	Y	18
Preis et al. [53]	Y	Y	Y	Y	Y	Y	Y	Y	Y	Y	20
Preis et al. [54]	Y	Y	?	Y	Y	Y	Y	Y	Y	Y	19
CASP items qualitative study checklist											
Bayes et al. [35]	Y	Y	Y	Y	Y	?	Y	Y	Y	Y	19
Borelli et al. [36]	Y	Y	Y	Y	Y	?	Y	Y	Y	Y	19
Colciago et al. [38]	Y	Y	Y	Y	Y	Y	Y	Y	Y	Y	20
Dahlen et al. [39]	Y	Y	Y	Y	Y	?	Y	Y	Y	Y	19
Fenwick et al. [41]	Y	Y	Y	Y	Y	?	Y	Y	Y	Y	19
Fleming [42]	Y	Y	Y	Y	Y	Y	Y	Y	Y	Y	20
Gibbins and Thomson [43]	Y	Y	Y	Y	Y	Y	Y	Y	Y	Y	20
Hauck et al. [44]	Y	Y	Y	?	Y	N	Y	Y	?	?	15
Hussein et al. [47]	Y	Y	Y	Y	Y	?	Y	Y	Y	y	19
Luyben et al. [49]	Y	Y	Y	Y	Y	?	Y	Y	Y	Y	19
Melender [49]	Y	Y	Y	Y	Y	?	Y	Y	Y	Y	19
Streibech and Ayerle [55]	Y	Y	Y	Y	Y	?	Y	Y	Y	Y	19
Turan et al. [56]	Y	Y	Y	Y	Y	?	Y	Y	Y	Y	19
Wicklund et al. [57]	Y	Y	?	Y	?	N	?	?	?	?	12

*Note*: CASP items: 1. Was there a clear statement of the aims of the research? 2. Is the methodology appropriate? 3. Was the research design appropriate to address the aims of the research? 4. Was the recruitment strategy appropriate to the aims of the research? 5. Was the data collected in a way that addressed the research issue? 6. Has the relationship between researcher and participants been adequately considered? 7. Have ethical issues been taken into consideration? 8. Was the data analysis sufficiently rigorous? 9. Is there a clear statement of findings? 10. How valuable is the research in the local context? Y = yes: Criterion completely met = 2 points:? = can't tell: criterion partially met = 1 point; *N* = no: criterion not applicable/unmet/not mentioned = 0 [21].

### 3.3 Epistemic Network Analysis (ENA)

We generated two models. We coded nine emotional touchpoints' content categories: *Coping Strategy*, *Process of Labor & Birth*, *Pain*, *Beliefs*, *Others*, *Birth Space*, *Procedures*, *Health*, and *Care*. The eight primary emotions were *Anger*, *Anticipation*, *Disgust*, *Fear*, *Joy*, *Sadness*, *Surprise*, and *Trust* [23]. Based on an agreement between the researchers, *Coping* is defined as conscious efforts and tactics used to manage labor and birth. *Process of Labor & Birth* are the actions or steps as part of labor and birth. *Pain* refers to labor pain, and *Beliefs* or belief systems are perceptions, thoughts, views, and ideas of labor and birth. *Others* refer to individuals being present and/or involved during labor and birth, and *Birth space* is the physical birth environment. *Procedures* refer to medical procedures, *Health* is the aspect relating to maternal and fetal/child health and *Care* are the activities and services needed to help and support the childbearing woman. *Anger* is regarded as a strong feeling of displeasure and antagonism. *Anticipation* is an expectation about something happening or coming. *Disgust* is a feeling of strong disapproval. *Fear* is an emotion caused by threat, danger, pain or harm. *Joy* is a feeling of pleasure and happiness. *Sadness* is regarded as unhappiness because of something bad or negative happening. *Surprise* is felt when encountering or discovering something unexpectedly or suddenly and *Trust* is having faith or confidence and believing in ability and reliability [23]. ENA identified the connections between emotional touchpoints and between the primary emotions, respectively. We set the moving stanza window size for the emotional touchpoints at nine lines because the papers included, on average, 8.7 entries and for the emotions at four lines because the papers included, on average, 3.9 entries. A simplified codebook of the emotional touchpoints and primary emotions per study and the coding is provided in Appendix B.

**FIGURE 2:**
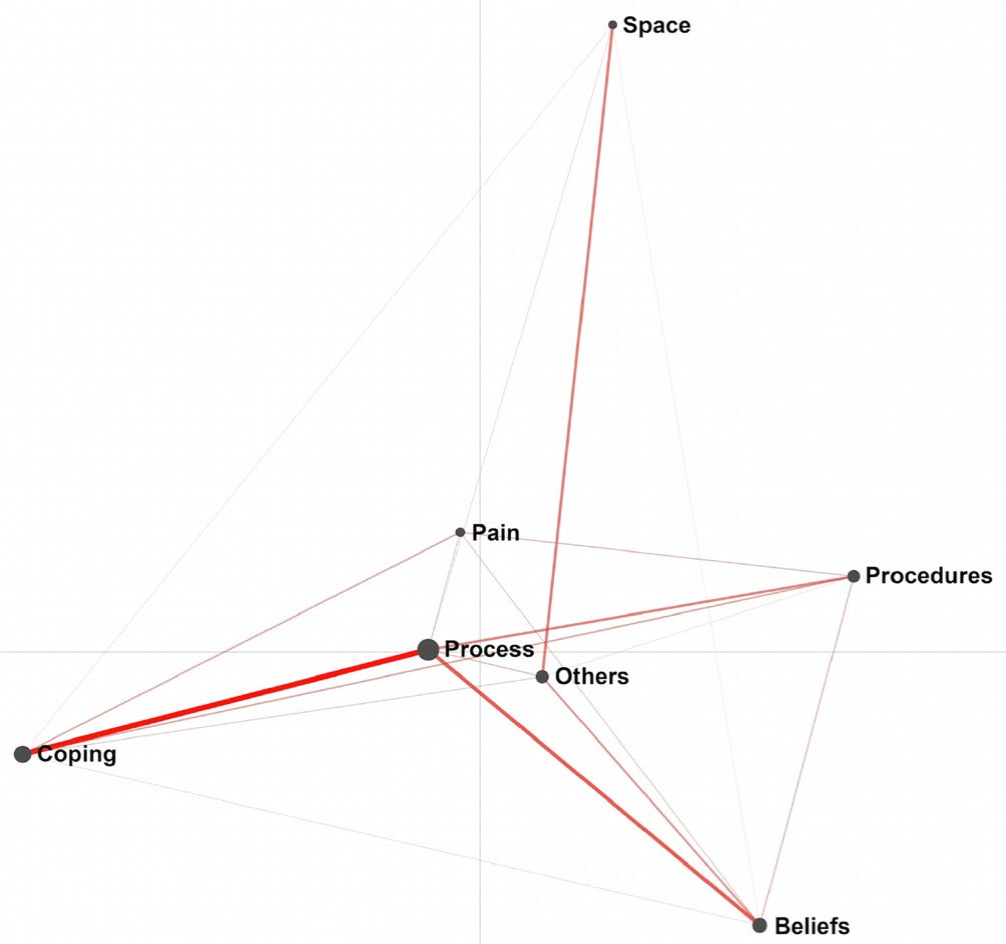
ENA model emotional touchpoints.

Figure 2 shows the model for the emotional touchpoints. Dimension 1 explained 20.2% of the variance (*r* 0.88), and dimension 2 explained 26.7% (*r* 0.89). The strongest connection was observed between *Coping* and *Process of Labor & Birth*. Examples of *Coping* are choice, control, sourcing information, mobility, staying calm, participation in decision‐making (pain relief) and debriefing. Examples of the *Process of Labor & Birth* are complications, the unknown, unexpected and unforeseen circumstances, interventions, mode of birth, the attitude of the care provider, and duration of the birth. The second strongest connection was observed between the *Process of Labor & Birth* and *Beliefs (belief systems) about Labor & Birth*. Beliefs relate to, for example, birth being a natural event or a medical event. There was a high co‐occurrence between the *Process of labor & Birth* and *Pain* (e.g., level of pain, pain relief, pain perception) and between *the Process of Labor & Birth* and *Others* (e.g., society, presence and relationship with partner or care professional). *Space* (e.g., place of birth, privacy, hygiene) was observed as an outlier. The reports originated from both nulli‐ and multiparous women obtained during pregnancy and the postpartum period.

**FIGURE 3:**
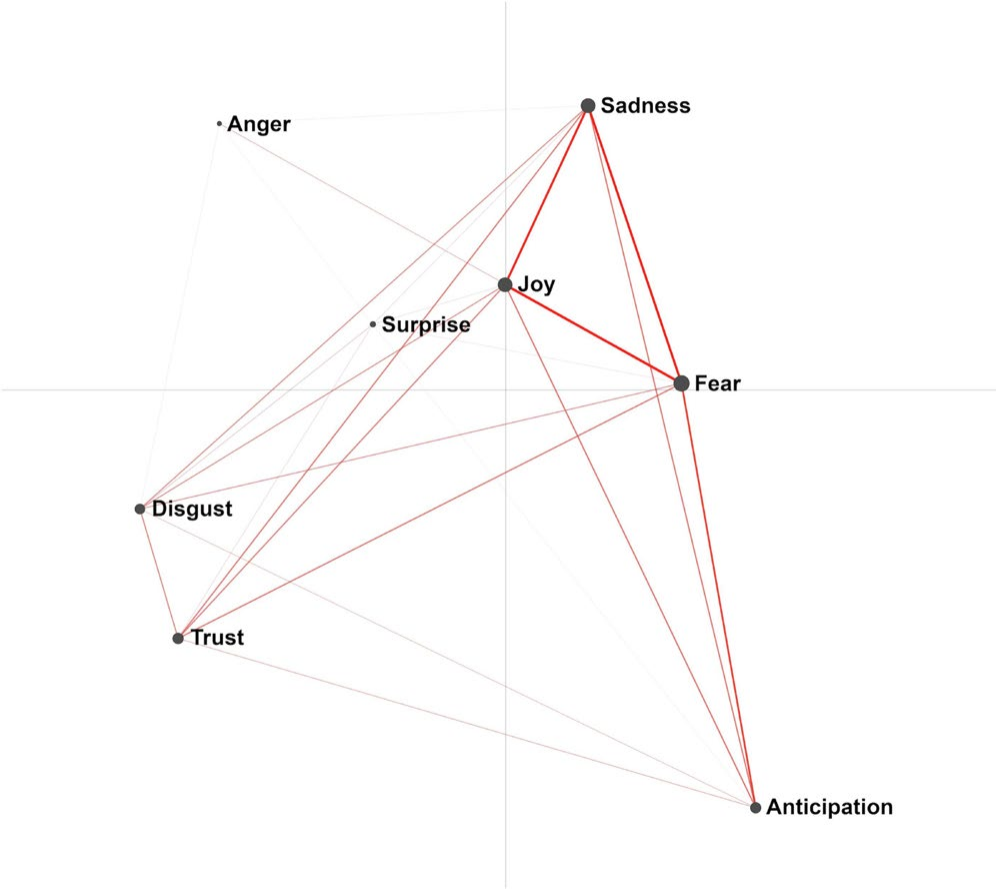
ENA model primary emotions.

Figure 3 shows the network model for the primary emotions. Dimension 1 explained 16.4% of the variance (*r* 0.91) and dimension 2 explained 42.3% (*r* 0.92). The equally strong connections with the highest co‐occurrence were between *Joy* and *Fear*, between *Fear* and *Sadness*, and between *Sadness* and *Joy*. Examples of *Joy* are happiness, feeling delighted, positive, satisfied, and fulfilled. Examples of *Fear* are anxiety, being afraid, frightened, and scared. Sadness was reported as, for example, disappointment, pain, or feeling cheated or exhausted. We observed *Anger* (e.g., feeling stupid or frustrated) and *Anticipation* (e.g., confidence, challenge, excitement) as outliers. The reports of *Joy* originated from nulli‐ and multiparous women and were mainly obtained during the postpartum period. Reports of *Fear* also originated from nulli‐ and multiparous women, reported during pregnancy and the postpartum period. Reports of *Sadness* originated more often from nulliparous women and were more often reported during the postpartum period.

## 4 Discussion

In this study, we explored the emotional touchpoints, adjectives, sentiments, and affective values in the reports of real‐life experiences and the experiential self‐reports of childbearing women. The emotional touchpoints and emotions we mapped shape how women experience and narrate birth. Our findings recognize childbirth as a profoundly emotional, embodied, and existential experience, rich in contradictory affects, supporting the perception that childbirth is not reducible to a single narrative or emotional outcome [58, 59]. From a feminist standpoint, this intermingling of fear and delight reflects the embodiment of childbirth [60]. Psychologically and phenomenologically, giving birth confronts women with mortality and the creation of life at once, inducing the co‐occurrence of opposite emotions—terror and joy, despair and relief [58, 59, 60]. The predominance of negative sentiments in our data (fear, sadness, loss) may itself reflect the biomedical discourse that frames birth as risky and pathological, an insight long noted in feminist critique [61].

From a salutogenic (health‐promotion) perspective, understanding childbirth through Antonovsky's Sense of Coherence helps explain why the organization of emotional touchpoints and emotions holds valuable. Antonovsky theorized that a strong sense of coherence involves perceiving life events—such as birth—as *comprehensible*, *manageable*, and *meaningful* [62, 63, 64]. Our ENA has distilled a complex affective field into structured domains and networks. This reduction—mapping multiple feelings into coherent clusters—can enhance the comprehensibility of the experience. It clarifies the “chaos” of birth by showing patterns and aligns with Antonovsky's idea that comprehensibility is a cornerstone of coping with birth [62, 64]. The ENA visualization may strengthen coherence as it provides women and healthcare providers with a map of emotional touchpoints, supporting a narrative that birth is an event that can be understood and managed, prospectively and retrospectively. This theorizes a pathway from emotional touchpoints and emotions to making sense of the emotional complexity of the birth event, aligning with salutogenic care [63, 64].

These theoretical connections have practical implications. Emotional literacy, the ability of caregivers to recognize and respond to women's feelings, is essential. In clinical practice, however, it seems impossible and unrealistic for care professionals to address all the emotional touchpoints and emotions when supporting women to prepare for or to debrief birth, or for women to consider all of them during pregnancy and the postpartum period [3, 4]. ENA facilitated the reduction of this multidimensionality to a set of connected emotional touchpoints: the process of labor and birth, women's beliefs about labor and birth, and coping with labor and birth and emotions: fear, joy and sadness. Reducing the multidimensionality may help to guide healthcare practitioners during antenatal and postnatal conversations with women, covering key topics, and preventing an overload of information [3]. Our findings indicate that the emotional touchpoints, framed in pregnant and postpartum women's real‐life experiences of childbirth, should be key topics of conversation in antenatal and postpartum dialogues between care professionals and childbearing women. Focusing on the three main categories of emotional touchpoints might offer a solution about which aspects of birth need to be seriously considered and discussed for women to develop meaningful, comprehensive, and manageable expectations and experiences [3, 4]. The words used by women to express emotions, presented in Table 1 and Appendix B, affirm that the emotional vocabulary women draw on to describe their birth experience is essential for sense‐making [65]. This emotional vocabulary might be helpful in conversations with women to support them in communicating their experiences, exploring emotions around a situation or experience and giving meaning to them [6, 15].

### 4.1 Strengths and Limitations

Data were extracted from an equal division of qualitative and quantitative studies, including a large sample of women, providing a more detailed and nuanced analysis than either study design alone, enhancing the robustness and reliability of the findings. We included international data, deriving from a large sample of childbearing women containing both nulliparous and multiparous women, enhancing the transformation of the findings to the childbearing community of women. As we are aware that differences can occur between parity [33], we were not able to differentiate and compare the findings of nulligravid/primiparous and multiparous women due to the considerable number of studies that included both groups, not allowing us to separate the findings of these groups of women. However, it could be beneficial to develop similar models for these subgroups as they require different conversations [33]. By performing two analyses, one of the emotional touchpoints and one of the primary emotions, we could not establish connections between specific touchpoints and the emotions. However, a nexus between the two models might exist, which could be explained by extracting the data from the same datasets of childbearing women, leading to similar predictions [66]. While some studies showed low quality, most showed moderate quality, and a few were classified as high quality. We amended the CASP Cohort Study tool according to Meekums and Daniel [17], excluding the two items which could have affected the quality appraisal of the quantitative studies. We modified the way emotional touchpoints are commonly used in research. In this methodology, one‐to‐one conversations or semi‐structured interviews are typically conducted, using adjectives to encourage a more in‐depth exploration of the experience [6]. Instead, we mapped and categorized reported outcome data from primary studies and analyzed the coded data. This could have introduced cognitive bias because coding relied on the researchers' interpretation, and therefore, we might have missed information. Our analysis did not explain the direction of the connections and maybe valuable information on the dynamics between elements was overlooked, requiring further research [67]. Also, we recognize that systematic reviews do not necessarily consider local contexts, affecting the contextual sensitivity of the results [68]. Despite the limitations of our study, using ENA has enabled two useful models of emotional touchpoints and emotions that highlight patterns and meaning in women's cognition [69]. It can be recommended to explore the use of the identified emotional touchpoints and emotions when these are used in dialogues with childbearing women.

## 5 Conclusions

This paper demonstrates how the complex array of birth‐related emotional touchpoints, along with the positive and negative emotions reported both prospectively and retrospectively by pregnant and postpartum women, were distilled into two distinct models. Our study recognizes childbirth as a profoundly emotional, embodied, and existential experience, rich in contradictory affects within a feminist and salutogenic perspective. The emotional touchpoints model emphasized that the labor and birth process, along with women's beliefs and coping strategies, are of great importance to them. The primary emotions model highlighted the emotions of fear, joy and sadness as relevant. Our findings provide care providers with information guiding the conversation surrounding birth, antenatal information and postpartum debriefing. The conversation should include the process of labor and birth, and delineation of beliefs or belief systems about labor and birth and sense‐making. Care providers should address the emotions of joy, fear, and sadness to support women in communicating, sharing their experiences and exploring their feelings. We found that ENA can be useful as an analytical method by providing meaning to the visual representations of childbearing women's emotional touchpoints related to birth and their birth‐related emotions.

## Conflicts of Interest

The authors declare no conflicts of interest.

## Supporting information


**Appendix A.** Inclusion criteria, search terms, search strategy and full database searches and results.

**Appendix B.** Sources, emotional touchpoints, emotions and coding. https://doi.org/10.5281/zenodo.13336033.
