# Supplementary material for: Emotional Touchpoints and Emotions of Childbirth: A Systematic Mixed Studies Review and Epistemic Network Analysis
Source: Perspect Sex Reprod Health. 2025 Jun 25;57(3):301–20. doi: 10.1111/psrh.70022 (PMC12421079; doi:10.1111/psrh.70022)
Supplement: Supplementary file 1 — Appendix A. Inclusion criteria, search terms, search strategy and full database searches and results. [file PSRH-57-301-s002.docx]

**Appendix A. Inclusion criteria, search terms, search strategy and full database searches and results**

| Inclusion criteria and search terms | | |
| --- | --- | --- |
| Criteria | Specification | Free text search terms |
| Population | Postpartum women (to three years postnatal).  Women of all parity, and modes of birth with a gestational age of 36 weeks or beyond and an age of 18 years or older.  High and low-risk women.  Women wo received care from any maternity care provider, either the midwife, obstetrician, or general practitioner, and who have given birth in any healthcare setting, either primary (including home), secondary, or tertiary. | Birth or antenatal or pregnancy or pregnant or postnatal or postpartum or prenatal or maternal or maternity or labour or childbirth or delivery or labor or pre birth or pre-birth or antepartum or perinatal or intrapartum or expectant mothers |
| Observation | The mismatch or incongruence between antenatal expectations with self-reported perceptions and experiences of the reality of birth.  The mismatch could refer to emotions felt during birth, birth preparedness, birth plan, fear, pain management. | Expectat* or perceptions or thoughts or opinions or desires or hopes or preferences or perceiv* or assumptions or assumed or beliefs or believed or conjecture or supposition or supposed or anticipat* or attitudes or ideal*  Reality or experience* or actual or actuality or happen* or occur* or reflect* |
| Outcome | (Retrospectively) reported postnatal emotions, satisfaction, sense of control or empowerment, choice and/or decision making | Incongruences or differ* or mismatch* or discrepancies or inconsisten* or conflict* or contradict* or discordan* or varian* or contrary or disparate or dissimilar or disparities or incompatible or dissonant or anomal* or opposites or discongru* or clash* or disagree* or dissidence or compar* or emotion or affect |
| Study design | Quantitative and qualitative primary research, including mixed methods studies involving any number of participants |  |

CINAHL Search undertaken 26^th^ May 2022

| Birth expectations | 1 | TI ( ( Birth or antenatal or pregnancy or pregnant or postnatal or postpartum or prenatal or maternity or labour or childbirth or labor or antepartum or perinatal or intrapartum or expectant) N2 ( Expectat* or perceptions or thoughts or opinions or hopes or assumptions or assumed or beliefs or believed or conjecture or supposition or supposed or anticipat* or ideal* ) ) OR AB ( ( Birth or antenatal or pregnancy or pregnant or postnatal or postpartum or prenatal or maternity or labour or childbirth or labor or antepartum or perinatal or intrapartum or expectant) N2 ( Expectat* or perceptions or thoughts or opinions or hopes or assumptions or assumed or beliefs or believed or conjecture or supposition or supposed or anticipat* or ideal* ) ) | 3,386 |
| --- | --- | --- | --- |
| Reality | 2 | (MH "Reflection") | 15,786 |
|  | 3 | TI ( Reality or experience* or actual or actuality or happen* or occur* or reflect* ) OR AB ( Reality or experience* or actual or actuality or happen* or occur* or reflect* ) | 947,166 |
|  | 4 | S2 OR S3 | 951,725 |
| Incongruences | 5 | TI ( Incongruence* or differ* or mismatch* or discrepanc* or inconsisten* or contradict* or discordan* or varian* or contrary or disparate or dissimilar or disparit* or incompatib* or dissonan* or anomal* or opposites or discongru* or clash* or disagree* or dissiden* or compar* ) OR AB (Incongruence* or differ* or mismatch* or discrepanc* or inconsisten* or contradict* or discordan* or varian* or contrary or disparate or dissimilar or disparit* or incompatib* or dissonan* or anomal* or opposites or discongru* or clash* or disagree* or dissiden* or compar* ) | 1,864,999 |
| All terms combined | 6 | S1 AND S4 AND S5 | 636 |
| Date from 2000 | 7 | S1 AND S4 AND S5 | 563 |
| Language Limited to English | 8 | S1 AND S4 AND S5 | 539 |

Medline Search undertaken 26^th^ May 2022

| Birth expectations | 1 | TI ( ( Birth or antenatal or pregnancy or pregnant or postnatal or postpartum or prenatal or maternity or labour or childbirth or labor or antepartum or perinatal or intrapartum or expectant) N2 ( Expectat* or perceptions or thoughts or opinions or hopes or assumptions or assumed or beliefs or believed or conjecture or supposition or supposed or anticipat* or ideal* ) ) OR AB ( ( Birth or antenatal or pregnancy or pregnant or postnatal or postpartum or prenatal or maternity or labour or childbirth or labor or antepartum or perinatal or intrapartum or expectant) N2 ( Expectat* or perceptions or thoughts or opinions or hopes or assumptions or assumed or beliefs or believed or conjecture or supposition or supposed or anticipat* or ideal* ) ) | 5,244 |
| --- | --- | --- | --- |
| Reality | 2 | TI ( Reality or experience* or actual or actuality or happen* or occur* or reflect* ) OR AB ( Reality or experience* or actual or actuality or happen* or occur* or reflect* ) | 4,165,276 |
| Incongruences | 3 | TI ( Incongruence* or differ* or mismatch* or discrepanc* or inconsisten* or contradict* or discordan* or varian* or contrary or disparate or dissimilar or disparit* or incompatib* or dissonan* or anomal* or opposites or discongru* or clash* or disagree* or dissiden* or compar* ) OR AB (Incongruence* or differ* or mismatch* or discrepanc* or inconsisten* or contradict* or discordan* or varian* or contrary or disparate or dissimilar or disparit* or incompatib* or dissonan* or anomal* or opposites or discongru* or clash* or disagree* or dissiden* or compar* ) | 11,280,395 |
| All terms combined | 4 | S1 AND S2 AND S3 | 921 |
| Date from 2000 | 5 | S1 AND S2 AND S3 | 782 |
| Language Limited to English | 6 | S1 AND S2 AND S3 | 745 |

PubMed Search undertaken 26^th^ May 2022

| Birth expectations | 1 | **Birth[Title/Abstract] OR antenatal[Title/Abstract] OR pregnancy[Title/Abstract] OR pregnant[Title/Abstract] OR postnatal[Title/Abstract] OR postpartum[Title/Abstract] OR prenatal[Title/Abstract] OR maternity[Title/Abstract] OR labour[Title/Abstract] OR childbirth[Title/Abstract] OR labor[Title/Abstract] OR antepartum[Title/Abstract] OR perinatal[Title/Abstract] OR intrapartum[Title/Abstract] OR expectant) N2 ( Expectat*[Title/Abstract] OR perceptions[Title/Abstract] OR thoughts[Title/Abstract] OR opinions[Title/Abstract] OR hopes[Title/Abstract] OR assumptions[Title/Abstract] OR assumed[Title/Abstract] OR beliefs[Title/Abstract] OR believed[Title/Abstract] OR conjecture[Title/Abstract] OR supposition[Title/Abstract] OR supposed[Title/Abstract] OR anticipat*[Title/Abstract] OR ideal*[Title/Abstract]** | 1,861,022 |
| --- | --- | --- | --- |
| Reality | 2 | **Reality[Title/Abstract] OR experience*[Title/Abstract] OR actual[Title/Abstract] OR actuality[Title/Abstract] OR happen*[Title/Abstract] OR occur*[Title/Abstract] OR reflect*[Title/Abstract]** | 4,197,869 |
| Incongruences | 3 | **Incongruence*[Title/Abstract] OR differ*[Title/Abstract] OR mismatch*[Title/Abstract] OR discrepanc*[Title/Abstract] OR inconsisten*[Title/Abstract] OR contradict*[Title/Abstract] OR discordan*[Title/Abstract] OR varian*[Title/Abstract] OR contrary[Title/Abstract] OR disparate[Title/Abstract] OR dissimilar[Title/Abstract] OR disparit*[Title/Abstract] OR incompatib*[Title/Abstract] OR dissonan*[Title/Abstract] OR anomal*[Title/Abstract] OR opposites[Title/Abstract] OR discongru*[Title/Abstract] OR clash*[Title/Abstract] OR disagree*[Title/Abstract] OR dissiden*[Title/Abstract] OR compar*[Title/Abstract]** | 11,322,192 |
| All terms combined | 4 | S1 AND S2 AND S3 | 156 |
| Date from 2000 | 5 | S1 AND S2 AND S3 | 138 |
| Language Limited to English | 6 | S1 AND S2 AND S3 | 138 |

Web of Science Core Collection Search undertaken 26^th^ May 2022

| Birth expectations | 1 | **( Birth or antenatal or pregnancy or pregnant or postnatal or postpartum or prenatal or maternity or labour or childbirth or labor or antepartum or perinatal or intrapartum or expectant) NEAR/2 ( Expectat* or perceptions or thoughts or opinions or hopes or assumptions or assumed or beliefs or believed or conjecture or supposition or supposed or anticipat* or ideal* )** (Title) or **( Birth or antenatal or pregnancy or pregnant or postnatal or postpartum or prenatal or maternity or labour or childbirth or labor or antepartum or perinatal or intrapartum or expectant) NEAR/2 ( Expectat* or perceptions or thoughts or opinions or hopes or assumptions or assumed or beliefs or believed or conjecture or supposition or supposed or anticipat* or ideal* )** (Abstract) | 6,754 |
| --- | --- | --- | --- |
| Reality | 2 | **Reality or experience* or actual or actuality or happen* or occur* or reflect*** (Title) or **Reality or experience* or actual or actuality or happen* or occur* or reflect*** (Abstract) | 7,276,044 |
| Incongruences | 3 | **Incongruence* or differ* or mismatch* or discrepanc* or inconsisten* or contradict* or discordan* or varian* or contrary or disparate or dissimilar or disparit* or incompatib* or dissonan* or anomal* or opposites or discongru* or clash* or disagree* or dissiden* or compar*** (Title) or **Incongruence* or differ* or mismatch* or discrepanc* or inconsisten* or contradict* or discordan* or varian* or contrary or disparate or dissimilar or disparit* or incompatib* or dissonan* or anomal* or opposites or discongru* or clash* or disagree* or dissiden* or compar*** (Abstract) | 21,090,637 |
| All terms combined | 4 | S1 AND S2 AND S3 | 1,070 |
| Date from 2000 | 5 | S1 AND S2 AND S3 | 973 |
| Language Limited to English | 6 | S1 AND S2 AND S3 | 907 |

Ovid Nursing Search undertaken 26^th^ May 2022

| Birth expectations | 1 | ((Birth or antenatal or pregnancy or pregnant or postnatal or postpartum or prenatal or maternity or labour or childbirth or labor or antepartum or perinatal or intrapartum or expectant) adj3 (Expectat* or perceptions or thoughts or opinions or hopes or assumptions or assumed or beliefs or believed or conjecture or supposition or supposed or anticipat* or ideal*)).ab,ti. | 6,754 |
| --- | --- | --- | --- |
| Reality | 2 | Reflection/ | 8,117 |
|  | 3 | (Reality or experience* or actual or actuality or happen* or occur* or reflect*).ab,ti. | 191,034 |
|  | 4 | 2 or 3 | 191,050 |
| Incongruences | 5 | (Incongruence* or differ* or mismatch* or discrepanc* or inconsisten* or contradict* or discordan* or varian* or contrary or disparate or dissimilar or disparit* or incompatib* or dissonan* or anomal* or opposites or discongru* or clash* or disagree* or dissiden* or compar*).ab,ti. | 278,935 |
| All terms combined | 6 | 1 and 4 and 5 | 217 |
| Date from 2000 | 7 | 1 and 4 and 5 | 187 |

Ovid Emcare Search undertaken 26^th^ May 2022

| Birth expectations | 1 | ((Birth or antenatal or pregnancy or pregnant or postnatal or postpartum or prenatal or maternity or labour or childbirth or labor or antepartum or perinatal or intrapartum or expectant) adj3 (Expectat* or perceptions or thoughts or opinions or hopes or assumptions or assumed or beliefs or believed or conjecture or supposition or supposed or anticipat* or ideal*)).ab,ti. | 2,345 |
| --- | --- | --- | --- |
| Reality | 2 | (Reality or experience* or actual or actuality or happen* or occur* or reflect*).ab,ti. | 1,255,638 |
| Incongruences | 3 | (Incongruence* or differ* or mismatch* or discrepanc* or inconsisten* or contradict* or discordan* or varian* or contrary or disparate or dissimilar or disparit* or incompatib* or dissonan* or anomal* or opposites or discongru* or clash* or disagree* or dissiden* or compar*).ab,ti. | 2,868,975 |
| All terms combined | 4 | 1 and 2 and 3 | 487 |
| Date from 2000 | 7 | 1 and 2 and 3 | 454 |
